# Undiagnosed Periprosthetic Infections in First-Time Aseptic Revision Hip Arthroplasties

**DOI:** 10.3390/biomedicines12102247

**Published:** 2024-10-02

**Authors:** Filippo Caternicchia, Francesco Castagnini, Danilo Donati, Bruno Cavalieri, Claudio Masetti, Michele Di Liddo, Giuseppe Tella, Francesco Traina

**Affiliations:** 1 Unità Operativa Ortopedia e Traumatologia, IRCCS Policlinico San Donato, 20097 San Donato Milanese, Italy; filippocaternicchia@virgilio.it; 2 SC Ortopedia-Traumatologia e Chirurgia Protesica e dei Reimpianti di Anca e Ginocchio, IRCCS Istituto Ortopedico Rizzoli, 40136 Bologna, Italy; bruno.cavalieri@ior.it (B.C.); claudio.masetti@ior.it (C.M.); michele.diliddo@ior.it (M.D.L.); giuseppe.tella@ior.it (G.T.); francesco.traina@ior.it (F.T.); 3 Physical Therapy and Rehabilitation Unit, Policlinico Universitario di Modena, 41122 Modena, Italy; danilo.donati@unimore.it; 4 Clinical and Experimental Medicine PhD Program, University of Modena and Reggio Emilia, 41121 Modena, Italy; 5 Dipartimento di Scienze Biomediche e Neuromotorie—DIBINEM, University of Bologna, 40127 Bologna, Italy

**Keywords:** periprosthetic infection, *Staphylococcus epidermidis*, C-reactive protein, intraoperative cultures, septic relapse, re-revision, low grade, occult, unexpected, single positive

## Abstract

**Background**: Unexpected infections diagnosed after intraoperative cultures in aseptic revision hip arthroplasties are infrequent, but the features and outcomes of culture-positive cases are still poorly understood. A single-center retrospective study was conducted to assess the following: (1) the incidence, (2) the profile of the cases, and (3) the outcomes of the revision hips performed for presumed aseptic reasons that became septic after intraoperative cultures. **Methods:** Instances of first-time aseptic revision hips (a retrospective cohort study) in the hospital database were reviewed. The revisions with the isolation of two phenotypically identical microorganisms in the intraoperative cultures were selected. The profile (bacteria, pre-operative markers) and the outcomes of the revisions (survival rates, complications, reasons for re-revision) were assessed. **Results:** Out of 424 cases of presumed aseptic revision hip arthroplasty, 19 patients (4.48%) were classified as septic. *Staphylococcus epidermidis* (9, 47.37%) was the most frequent microorganism. In three patients (15.8%), C-reactive protein and erythrocyte sedimentation rate values were higher, and in only one case (5.26%), C-reactive protein values and the white blood cell count were elevated. An antibiotic therapy was administered in every case. At a mean follow-up of 3.72 ± 2.18 years, three patients (15.79%) experienced complications (dislocation, pain without loosening, chronic suppressive antibiotic therapy) and two patients (10.53%) required re-revision for septic relapse (same microorganisms). The survival rate of the cohort was 89.47% (95% CI: 64.08–97.26) at 2 and 4 years. **Conclusions:** Missed periprosthetic infections rarely occurred in presumed aseptic revision hips. However, the outcomes are fair, and septic relapses are not uncommon.

## 1. Introduction

The number of revision hip arthroplasties is steadily increasing over time and is expected to account for 85,528 procedures by 2030 in United States [1]. Aseptic loosening and periprosthetic hip infection (PHI) are the two main reasons for revision [1]. Although the current guidelines for PHI diagnosis (2018 Definition of Periprosthetic Hip and Knee Infection) demonstrated a higher sensitivity (97.7%) compared to the Musculoskeletal Infection Society (79.3%) and International Consensus Meeting (86.9%) recommendations, some cases of PHI are clinically misdiagnosed and identified only in retrospect after intraoperative cultures [2]. In a Denmark registry study, unexpected positive cultures were detected in 12% of revisions performed for clinically presumed aseptic loosening, and 5% of the cases had two or more positive cultures identifying the same microorganism [3]. In these cases, the etiology of infection can be mainly attributed to low-virulence microorganisms, especially Coagulase-negative *Staphylococci*, surviving on metal surfaces in favorable biofilms and possibly causing secondary implant loosening [4]. The treatment and outcomes of missed PHI are contradictory. Oral or intravenous antibiotic treatments were preferentially administered for 4–6 weeks in many case series, while some authors reported longer therapies or prescribed no antibiotics at all [5,6]. The outcomes of unexpected PHI seemed favorable in most case series, with survival rates of around 89–100% [5,6,7,8,9,10]. However, some case series reported much lower outcomes, with up to 50% of re-revisions [5,6,7,8,9,10]. However, much of the current knowledge about misdiagnosed PHI comes from small case series, with mixed populations of hip and knee arthroplasties, old diagnostics, and different therapeutic strategies [5,6,7,8,9,10]. Among the largest cases series, Saleh et al. investigated the outcomes of 103 unsuspected PHI, but adopted different classifications and treatment strategies (with and without short-term antibiotic courses) [6]. Similarly, Jacobs et al. identified 17 unsuspected periprosthetic knee infections and 26 unsuspected PHI, but, once again, no predefined therapeutical criteria were adopted [7]. Neufeld et al. identified 35 two-culture-positive unsuspected PHI but adopted different antibiotic strategies [8]. Vargas-Reveron et al. identified 13 two-culture-positive PHI that were treated with a targeted antibiotic therapy for at least four weeks; the authors reported a re-revision rate of 23.1% at a mean follow-up of four years [10].

Considering the limits of the current knowledge, we therefore conducted a single-center retrospective study to evaluate the revision hips performed for presumed aseptic reasons that turned out to be misdiagnosed PHI after intraoperative positive cultures. We sought to assess the following: (1) the incidence of unsuspected PHI in revision hip arthroplasties performed for presumed aseptic reasons; (2) the demographics of these cases, the implant features, and the involved microorganisms; and (3) the outcomes of these cases and the reasons for re-revision. We hypothesized that 5% of unsuspected PHI could be detected, with a survival rate higher than 80% after single-stage revision and targeted antibiotic therapy.

## 2. Materials and Methods

The institutional review board approved the study protocol (349/2021/Oss/IOR, 10 May 2021). A single-center retrospective prognostic study was performed, reviewing the institutional database for all cases of patients undergoing first-time revision hip arthroplasties for presumed non-septic reasons between January 2011 and December 2018.

The inclusion criteria were as follows: adult patients, first-time revision hip arthroplasty, non-septic reasons for revision, no medical history of PHI before the index revision surgery, at least five intraoperative samples for culture examination, and a minimum 2-year follow-up after the revision surgery.

The exclusion criteria were as follows: results of intraoperative cultures not available or incomplete, PHI diagnosis before revision surgery, follow-up inferior to 2 years, or antibiotic therapy within 14 days before revision surgery.

All the revisions shared the same pre-operative diagnostic work-up to rule out PHI; this work-up was partially developed on the 2019 Infectious Diseases Society of America consensus [11]. It consisted of anamnesis, physical examination, serial plain radiographs, serial laboratory tests with synovial C-reactive protein (CRP) and erythrocyte sedimentation rate (ESR) markers, CT scan, and triple-phase bone scintigraphy. In cases of doubtful, inconclusive, or suspected findings, 18-fluorodeoxyglucose positron emission tomography or white blood cell scintigraphy and ultrasound-guided synovial fluid aspiration were performed. In the synovial fluid, microbiological cultures and synovial fluid biomarkers (white blood cell count, polymorphonuclear leukocytes count, CRP) were assessed.

Pre-operatively, all the patients received a single intravenous administration of 2 g cefazolin for antibiotic prophylaxis (alternatively, clindamycin 600 mg was administered in case of allergy). The prophylaxis was discontinued in the perioperative setting. The intraoperative work-up required at least five periprosthetic tissue samples for culture examination. The sampling technique required the excision of non-necrotic vital tissues. A synovial fluid sample was collected just before the capsular incision. The sonication of removed implants was performed at the surgeon’s discretion, according to the method described in a previous paper [12]. Tissue samples for culture were sent to the same microbiology laboratory, where they were processed in a standard fashion in aerobic and anaerobic cultures incubated at 35 °C.

### 2.1. Unsuspected PHI Definition

The 2018 definitions of PHI were adopted [2]. A retrospective diagnosis of unsuspected PHI was made when at least two cultures identified the same pathogen. A single positive specimen, even isolating a low-virulent, slow-growing bacterium, was not considered.

### 2.2. Study Population, Treatment, and Outcomes

The demographics, the comorbidities, and the implant features of the selected cases were collected. The identified bacteria were recorded, as well as the antibiotic resistances. The eventual antibiotic therapy was prescribed by the infectious disease consultant.

The patients were prospectively followed until the date of death or the last available follow-up. Chronic suppressive antibiotic therapy, the presence of a fistula, and revision procedures were considered failures.

### 2.3. Statistical Analysis

Demographic information, comorbidities, implant-related features, and reasons for re-revision were presented as raw data, ranges, and percentages. The survival rates were calculated using the Kaplan–Meier technique, using 95% confidence intervals. The curve was considered reliable as long as at least 25% of the implants were still at risk at the established follow-up. The statistical analysis was performed using SPSS 14.0 for Windows, version 14.0.1 (SPSS, Chicago, IL, USA).

## 3. Results

### 3.1. Incidence

A total of 424 first-time revision hips performed for non-septic reasons in 424 patients matched the inclusion criteria and were identified. Out of these cases, 65 (15.33%) showed a single positive culture, of which 21 (5%) also had a pre-operative elevated CRP. Two patients (0.47%) presented two positive cultures, but the isolated microorganisms were not phenotypically identical.

Out of the 424 cases of presumed non-septic revision hip arthroplasty, 19 patients (4.48%) were classified as unsuspected PHI.

### 3.2. Demographics, Implant-Related Features, and Isolated Microorganisms

The demographics and the implant-related features of the cohort are detailed in Table 1.

The Charlson Comorbidity Index for estimating 10-year survival in patients with multiple comorbidities was five or more in one patient (5.26%), who was also affected by chronic renal insufficiency. One patient (5.26%) presented with obesity with a BMI > 30 kg/m^2^. None of the patients in the cohort were taking immune-suppressive drugs or were affected by immune-suppressive disorders. Among comorbidities sustaining PHI, two patients (10.53%) had non-insulin-dependent (type II) diabetes, and one patient (5.26%) had end-stage kidney disease. No other relevant comorbidities were recorded, especially immunosuppression, hepatic cirrhosis, or neoplastic conditions. The elapsed time between THA and revision was 10.78 ± 5.76 years (range: 1–21).

The mean pre-operative value for ESR was 31.67 ± 17.46 mm, range: 9–86 (threshold: 36 mm). The mean pre-operative value for CRP was 1.07 ± 1.55 mg/dL, range: 0.1–5.71 (threshold: 0.5 mg/dL). The mean pre-operative white blood cell count in the serum was 8.87 ± 2.45 × 103 per mcL, range: 5.9–15.23 (threshold: 11 × 103 per mcL). Eleven patients (57.89%) showed elevated pre-operative ESR. Only five patients (26.32%) were reported to have higher CRP values, and two patients (10.53%) had higher white blood cell counts. In three patients (15.8%), CRP and ESR values were higher, and in only one case (5.26%), CRP and the white blood cell count in the serum were elevated. In two cases (10.53%), synovial fluid aspiration was performed, but the white blood count, polymorphonuclear percentage, and synovial CRP were not elevated and the cultures were negative.

*Staphylococcus epidermidis* (9, 47.37%) was the most frequently isolated microorganism (Table 2).

In one patient (5.26%), polymicrobial growth was traced (*Staphylococcus epidermidis* and Coagulase Negative *Staphylococcus*). Bacteria resistances are shown in the Supplementary Materials.

### 3.3. Outcomes

Thirteen cases (68.42%) required isolated revisions of acetabular or femoral components. In two cases (10.53%), the whole implant was revised. In four cases (21.05%), only the modular components were exchanged. No antibiotic-loaded component was implanted. A microorganism-specific 4–6-week antibiotic therapy was administered after cultures in every case, according to resistance and patients’ comorbidities; the prescriptions were made by an infectious disease consultant. The mean follow-up of revised cases was 3.72 ± 2.18 years (range: 2–6 years). Three patients (15.79%) experienced complications: a conservatively treated dislocation, persistent pain without signs of loosening, and a chronic suppressive antibiotic therapy (*Staphylococcus haemolyticus* and *Staphylococcus epidermidis* were isolated). Two patients (10.52%) required re-revision for septic relapses; the same microorganisms sustained the septic relapses (*Staphylococcus lugdunensis* and *Staphylococcus epidermidis*). These cases were treated with a two-stage approach (after two years). The survival rate of the cohort was 89.47% (95% CI: 64.08–97.26) at two and four years (considering chronic suppressive antibiotic therapy as a failure).

## 4. Discussion

Revisions for presumed aseptic reasons revealed missed PHI with at least two identical positive cultures in 4.48% of cases. The most frequently involved microorganism was *Staphylococcus epidermidis*. In these cases, the pre-operative work-up did not anticipate the diagnosis or revealed only minor serological alterations. All the cases were treated with cementless revisions. After the culture findings, an antibiotic therapy was administered for 4–6 weeks. At a mean follow-up of 3.72 ± 2.18 years, three patients (15.79%) experienced complications, and two patients (10.53%) required a re-revision for septic relapse, sustained by the same microorganism. The survival rate of the cohort was 89.47% at four years. The hypotheses were confirmed.

This study has many limits: the retrospective design, the small number of cases, and the short follow-up. The use of conventional cultures, instead of next-generation sequencing techniques, should not have reduced the validity of the present study, according to the current literature [13]. Some patient-related factors that can increase the risk of prosthetic infection, like previous prolonged hospitalization or hospitalization in a nursing home, significant blood loss or blood transfusion, excessive tissue trauma, the presence of nosocomial bacterial strains, or failure to follow the rules of asepsis and antisepsis, have been partially collected because many primary THA were performed outside our hospital [14]. On the other side, strict selection criteria were applied, limiting the cases to first-time revision hip arthroplasties with two positive cultures. The systematic pre-operative work-up gave a true perspective on the percentage of missed PHI in first-time aseptic revision hip arthroplasties. Moreover, for all the cases, a similar treatment was adopted, with no antibiotic-loaded cemented component revision and 4 to 6-week antibiotic therapy, providing a consistent view on the outcomes of a single-treatment strategy.

The most frequently isolated microorganism was *Staphylococcus epidermidis*, followed by Coagulase Negative *Staphylococcus*. *Staphylococcus epidermidis* is a Coagulase negative *Staphylococcus*, a commensal of the skin flora, but it also acts as an opportunist pathogen [15]. It is a biofilm producer and caused one-third of periprosthetic joint infections [15,16,17,18]. The previous studies about unexpected PHI recognized *C. Acnes* and Coagulase Negative *Staphylococcus* as the most frequent bacteria [4]. All these bacteria are commensal of the skin, tend to have opportunistic behaviors, produce biofilms, and may cause silent low-grade infections with minimal serological alterations [19,20].

As a matter of fact, in the current study, there was a small fraction of cases showing alteration of the inflammatory serum markers (around one-fourth). This finding was also highlighted by Neufeld et al., recording similar percentages [6]. Nevertheless, Hipfl et al. noticed higher rates of elevated CPR, with a significant correlation between higher values and low-grade infections (predominant bacteria: Coagulase Negative *Staphylococci*) [21]. Moreover, both the case series highlighted that 20% of the cases underwent pre-operative synovial fluid aspiration, without success (in the present report: 10%) [6,21].

The survival rates of revised implants with two positive cultures were fair at short-to-midterm follow-ups. These outcomes were similar to those reported in the literature for unsuspected PHI, although the diagnostic criteria were not always consistent among the studies. In a systematic review assessing the survival rates of missed periprosthetic joint infections (hip and knee arthroplasties), only a few papers reported unsatisfying outcomes, with many septic re-revisions. Staat et al. identified a re-revision rate of 30% [9]. In general, presumed aseptic revisions of missed PHI showed outcomes substantially similar to aseptic revision hips, with or without single-positive cultures [5,6,22].

Among the reasons for re-revision, PHI represented a large portion of re-interventions. In the present reports, all the re-revisions were due to septic recurrence. The possible influence of the lack of antibiotic-loaded cement in revision procedures could be argued, which is a solution that was frequently adopted in other case series [6,22]. However, it should be noted that in some cases (Boot et al.), chronic infections not leading to another surgical treatment were not classified as re-revisions. It is likely that infection recurrence could be understated (and survival curves overstated), at least in very large cohort studies [20,21,22,23,24]. Neufeld et al. noticed that a septic relapse sustained by the first-time microorganism was a frequent reason for re-revision [6]. This finding was confirmed in the present report, with all the re-revisions being due to the same microorganism isolated in the first revision. However, any conclusions about septic recurrence are merely speculative due to the very low numbers provided by the current case series.

## 5. Conclusions

In summary, first-time revision hips with two positive intraoperative cultures are difficult to detect in the pre-operative setting and are usually sustained by low-virulent bacteria. These cases, when treated with antibiotic therapy, may achieve acceptable performances at short-to-midterm follow-ups. However, septic relapses may occur and are probably the main reason for re-revisions. While the rare occurrence of this condition does not allow for any strong conclusions about the best treatment strategy and the possible outcomes, a single-stage partial revision, even with non-antibiotic-loaded cemented components followed by an antibiotic therapy, may be sufficient, and general positive outcomes should be expected.

## Figures and Tables

**Table 1:** Unsuspected PHI population (n = 19), demographic and clinical details. ASA, American Society of Anesthesiologists; CoC, ceramic-on-ceramic; CoP, ceramic-on-polyethylene; MoM, metal-on-metal; MoP, metal-on-polyethylene; THA, total hip arthroplasty.

Demographics and Implant Features	Data
**Mean age at THA, yrs (range)**	48.78 ± 13.65 (27–76)
**Mean age at THA revision, yrs (range)**	60.32 ± 13.81 (30–84)
**Mean weight (kg)**	73.84 ± 16.38 (51–118)
Female sex, n (%)	13 (68.42)
Male sex, n (%)	6 (31.58)
**Mean BMI, kg/m^2^ (range)**	25.72 ± 3.69 (21.5–36)
Overweight patients	8 (42.11%)
Obese patients	1 (52.63%)
**ASA score, n (%)**	
1 and 2	15 (78.95)
3 and 4	3 (15.79)
Missing data	1 (5.26)
**Charlson Comorbidity index, n (%)**	
0–2	14 (76.68)
3–4	4 (21.05)
5 or more	1 (5.26)
**Smoking patients, n (%)**	3 (15.79%)
**Primary THA fixation, n (%)**	
Uncemented	17 (89.47)
Cemented	0 (0)
Hybrid	2 (10.53)
**Primary THA bearings, n(%)**	
CoP	7 (36.84)
MoP	7 (36.84)
MoM	2 (10.53)
CoC	3 (15.79)
Missing data	0 (0)
**Reasons for revision, n (%)**	
Cup loosening	12 (63.15)
Stem loosening	3 (15.78)
Global loosening	2 (10.52)
Neck breakage	1 (5.26)
Osteolysis and periprosthetic hip fracture	1 (5.26)

**Table 2:** Isolated microorganisms in 19 low-grade infections.

Isolated Microorganisms	Patients with Two Positive Cultures at Least (%)
*Staphylococcus epidermidis*	9 (47.37) *
Coagulase Negative *Staphylococci*	3 (15.79) *
*Staphylococcus hominis*	2 (10.53)
*Staphylococcus haemolyticus*	2 (10.53)
*Staphylococcus warneri*	2 (10.53)
*Enterococcus faecium*	1 (5.26)
*Staphylococcus lugdunensis*	1 (5.26)

* The sum of isolated microorganisms exceeds the number of patients because a revision had polymicrobial growth with two cultures of *Staphylococcus epidermidis* and two cultures of Coagulase Negative *Staphylococci*.

## Data Availability

Data are contained within the article.

## Supplementary Materials

The following supporting information can be downloaded at https://www.mdpi.com/article/10.3390/biomedicines12102247/s1, Bacteria resistances.
