# Supplementary material for: Undiagnosed Periprosthetic Infections in First-Time Aseptic Revision Hip Arthroplasties
Source: Biomedicines. 2024 Oct 2;12(10):2247. doi: 10.3390/biomedicines12102247 (PMC11504665; doi:10.3390/biomedicines12102247)
Supplement: Supplementary file 1 [file biomedicines-12-02247-s001.zip › biomedicines-3223019-supplementary.pdf]

Bacteria resistance of the 19 2-culture positive cases

n= Patient , Tot= 19	Clarithromycin	Clindamycin	Erythromycin	Gentamicin	Netilmicin	Penicillin	Ampicillin	Chloramphenicol	Levofloxacin	Fosfomycin	Tetracycline	Ciprofloxacin	Oxacillin	Moxifloxacin	Rifampicin	Trimet-sulfamethoxazole
1- Staphylococcus epidermidis	R	R	R	R	R	R										
2- Enterococcus faecium			R				R	R	R	R	R					
3- Staphylococcus epidermidis ;Coagulase-negative staphylococci									R			R	R			
									R			R		R		
4-Coagulase-negative staphylococci	R		R			R										
5- Staphylococcus epidermidis						R										
6- Staphylococcus warneri						R				R						
7- Staphylococcus epidermidis	R	R		R	R	R		R	R		R	R	R	R	R	R
8- Staphylococ-				R	R	R										

**cus epidermidis**																
**9-Staphylococcus Haemolyticus**		**R**														
**10-Staphylococcus epidermidis**	**R**		**R**	**R**	**R**	**R**										
**11- Staphylococcus lugdunensis**	**R**	**R**	**R**			**R**	**R**									
**12-Staphylococcus Haemolyticus**	**R**		**R**													
**13-Staphyloccoccus hominis**											**R**					
**14-Coagulase-negative staphylococci**																
**15-Staphylococcus epidermidis**	**R**	**R**	**R**			**R**							**R**			
**16-Staphylococcus epidermidis**	**R**			**R**	**R**	**R**										
**17-Staphylococcus hominis**											**R**					
**18-**				**R**	**R**	**R**										

**Staphylococcus epidermidis**																
**19-Staphylococcus warneri**						**R**				**R**						
